# Let-7 in Cardiovascular Diseases, Heart Development and Cardiovascular Differentiation from Stem Cells

**DOI:** 10.3390/ijms141123086

**Published:** 2013-11-21

**Authors:** Mei-Hua Bao, Xing Feng, Yi-Wen Zhang, Xiao-Ya Lou, Yu Cheng, Hong-Hao Zhou

**Affiliations:** 1 Institute of Clinical Pharmacology, Central South University, Changsha 410078, China; E-Mails: mhbao78@163.com (M.-H.B.); zjzyw2003@163.com (Y.-W.Z.); lxy_csu2013@163.com (X.-Y.L.); csuicp@163.com (Y.C.); 2 Department of Pharmacy, Changsha Medical University, Changsha 410219, China; 3 College of Medicine, Hunan Normal University, Changsha 410006, China; E-Mail: fengxing01_2013@163.com

**Keywords:** let-7, cardiovascular disease, cardiovascular differentiation, heart development, biomarker

## Abstract

The let-7 family is the second microRNA found in *C. elegans*. Recent researches have found it is highly expressed in the cardiovascular system. Studies have revealed the aberrant expression of let-7 members in cardiovascular diseases, such as heart hypertrophy, cardiac fibrosis, dilated cardiomyopathy (DCM), myocardial infarction (MI), arrhythmia, angiogenesis, atherosclerosis, and hypertension. Let-7 also participates in cardiovascular differentiation of embryonic stem cells. TLR4, LOX-1, Bcl-xl and AGO1 are by now the identified target genes of let-7. The circulating let-7b is suspected to be the biomarker of acute MI and let-7i, the biomarker of DCM. Further studies are necessary for identifying the gene targets and signaling pathways of let-7 in cardiovascular diseases. Let-7 might be a potential therapeutic target for cardiovascular diseases. This review focuses on the research progresses regarding the roles of let-7 in cardiovascular development and diseases.

## 1. Introduction

MicroRNAs (miRNA) are small, endogenous, noncoding RNAs, consisting of 21–25 nucleotides. These small single strand miRNAs target one or more mRNAs, and regulate the expression of the target gene by degradation, translation inhibition, or translational activation of their target mRNA [1,2]. It has been reported that miRNAs may directly regulate roughly one third of the genes in a cell [3].

The first miRNA, lin-4, was discovered in 1993 in *C. elegans* [4]. Let-7 was the second miRNA found also in *C. elegans*. Both lin-4 and let-7 regulate the development of *C. elegans* [5]. For the time being, more than 2000 miRNAs have been discovered in humans [6].

The human let-7 family contains 13 members located in 9 chromosomes. These members are respectively let-7a-1, let-7a-2, let-7a-3, let-7b, let-7c, let-7d, let-7e, let-7f-1, let-7f-2, let-7g, let-7i mir-98, and mir-202 [7]. Some of them are located in genome as clusters. For example, let-7a-1, let-7d and let-7f-1 form the intergenic cluster mapped to chromosome 9q22.32, whereas let-7b and let-7a-3 form the intergenic cluster mapped to chromosome 22q13.31.

**Figure 1:**
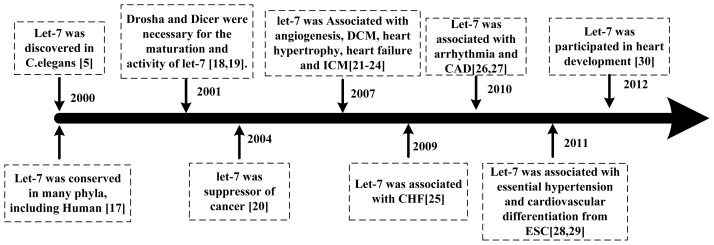
The timeline of let-7 discovery in cardiovascular diseases.

Many studies have identified let-7 as a tumor suppressor, which is down-regulated or lost in many human cancers. The restoration of let-7 expression may be a useful therapeutic option for cancers [8,9]. More recently, the roles of let-7 in cardiovascular biology and disease have received significant attentions (Figure 1). Let-7 have been found to be highly expressed in all major types of cardiovascular cells, including vascular smooth muscle cells (VSMC) [10], endothelial cells (EC) [11], cardiomyocytes [12–14], and coronary arterial smooth muscle cells [15]. Particularly for ECs, different let-7 levels have been observed in different EC types, such as HUVEC, human brain microvascular endothelial cell (HBMVEC), human coronary endothelial cells (HCECs), and human pulmonary artery endothelial cells (HPAECs), suggesting that let-7 contribute to the unique phenotypic diversity of ECs [16].

The let-7 family has been discovered to play important roles both in cardiovascular biological procedures and cardiovascular diseases. It acts as a switch and regulator in the development, functions and diseases of cardiovascular system. Although the precise roles of let-7 in these processes are unclear, more and more its potential target genes involved in cardiac signaling and transcription pathways have become apparent during the last years. Here, we review the research progress regarding the roles of let-7 in cardiovascular development and diseases.

## 2. Let-7 in Cardiovascular Diseases

### 2.1. Let-7 in Cardiac Hypertrophy, Heart Development and Cardiac Fibrosis

Let-7 was first identified as an essential developmental gene in *C. elegans*, and was demonstrated to be a regulator for stem-cell differentiation in *C. elegans*, as well as cell proliferation and differentiation in cancer cells. It can thus be reasonable to hypothesize that they may play important roles in cardiac hypertrophy, heart development and heart fibrosis. This hypothesis of let-7 has been clarified by several researches both *in vitro* and *in vivo* [31].

Cardiac hypertrophy is a pathological manifestation in chronically stressed hearts and is a significant factor in the pathogenesis of heart failure. In the pathogenesis of hypertrophy, complex signaling networks have been discovered in the latest decades. Recently, the role of miRNAs in cardiogenesis has received great attention. The miR-1, miR-133, miR-126 and miR-143/145 have been confirmed to parcitipate in the cardiac development and hypertrophy [32,33]. A commonly used mouse model of postnatal hypertrophy, transverse aortic constriction (TAC), was adopted by the Sayed D group to investigate miRNAs on postnatal cardiac pathophysiology. They found a complex array of miRNAs is dysregulated, including let-7d been down-regulated and let-7b/c been up-regulated after 14 days of TAC [21]. A very recent study by Yang *et al.* on pressure-overloaded or angiotensin II treated hearts and isolated cardiomyocytes reported that miR-98 along with other let-7 members were up-regulated [34,35]. Since pressure-overload and angiotensin II are two crucial factors in heart hypertrophy, the results imply that miR-98 and let-7 might play important roles in heart hypertrophy. Although the precise function and signal pathway of let-7 in cardiac hypertrophy is still unclear, the role of a redox- dependent protein, Thioredoxin 1 (Trx1), has been recognized. Trx1 suppresses cardiac hypertrophy by modifying histone deacetylase (HDAC) class II and apoptosis signal-regulating kinase [36,37]. MiR-98 is downstream effector of Trx-1 with potential importance, and cyclin D2 was a functional target of miR-98. Therefore, the Ang II/Trx-1/miR-98/Cyclin D_2_ signaling pathway form a negative-feedback loop to suppress Ang II induced heart hypertrophy [34,35].

Cardiac fibrosis is a common end-stage pathologic manifestation of several cardiovascular diseases. Fibroblasts are the major sources of extracellular matrix (ECM) during cardic fibrosis. The origin of resident cardiac fibroblasts is thought to be derived from embryonic mesenchymal cells in response to myocardial infarction. Recent studies suggest that adult fibroblast-like cells, originated from endothelial cells by endothelial-to-mesenchymal transition (EndMT), contribute to the pathogenesis of cardic fibrosis [38,39]. MiRNA array data reveals that let-7c and let-7g along with other miRNAs are significantly elevated during EndMT [40], indicating that let-7 might take part in EndMT.

### 2.2. Let-7 in Myocardial Infarction (MI) and Heart Failure

MI is a severe disease causing cardiac injuries and even heart failure. To investigate whether let-7 members participate in MI and heart failure, several studies have been undertaken. An *in vitro* study demonstrated that let-7f was up-regulated in myocardial ischemia [26], and an *in vivo* study found let-7c was up-regulated in biopsy specimens of 67 patients diagnosed as suffering aortic stenosis, ischemic cardiomyopathy (ICM), or idiopathic cardiomyopathy [22]. A “group analysis” was executed by Shi *et al.* in 2009 [25]. They found that let-7 members were up-regulated at the significant FDR (false discovery rate) of 14%, confirming the relationship between the let-7 family (let-7b/7c/7d/7e/miR-98) and congestive heart failure (CHF) [25]. These findings suggest that let-7 might be an active role in the pathogenesis of MI and heart failure. However, the effects and mechanisms of let-7 on MI and heart failure are still to be investigated in further studies.

Interestingly, Thum *et al.* [23] found the let-7c expression alterations were strikingly similar in failing human hearts and fetal hearts (2.2 folds in failing hearts and 2.9 folds in fetal hearts compared with healthy hearts respectively). Moreover, transfection of cardiomyocytes with a set of fetal miRNAs induced cellular hypertrophy as well as changes in gene expressions similar to those in the failing heart. Since chronic heart failure is characterized by reactivation of a fetal gene program, this research demonstrates that miRNAs (including let-7) might be responsible for the gene expression changes in failing heart. Development of drugs and molecules specifically regulating cardiac miRNAs (including let-7) followed by the subsequent normalization of altered target expression may lead to novel treatments for heart failure [23].

### 2.3. Let-7 in Dilated Cardiomyopathy (DCM)

**Figure 2:**
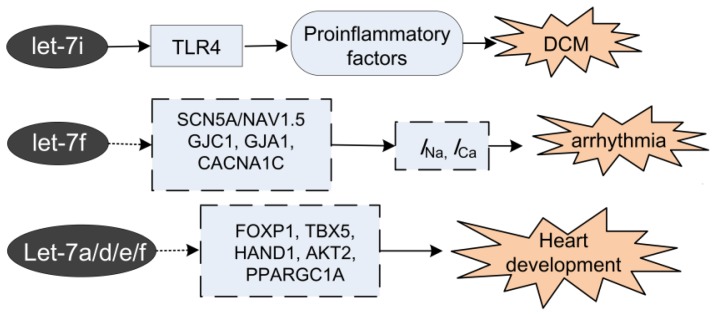
Targets of let-7 in dilated cardiomyopathy (DCM), arrhythmia and heart development. The solid arrow indicates the confirmed targets and the dotted arrow indicates the predicted targets of let-7.

DCM is the most common form of cardiomyopathy associated with heart failure. During the pathogenesis of DCM, toll-like receptors 4 (TLR4) has been found to be an important participator. TLR4 leads to the expression of nuclear factor κB, and is involved in the downstream of key pro-inflammatory cytokines in the myocardium from DCM patients [41]. It has been shown that let-7i target TLR4 at the post-transcription stage and limits the expression of this receptor [24]. *In vivo* study on DCM patients confirmed the negative relationship of let-7i and TLR4. In the study, the expression between let-7i was down-regulated in DCM patients compared with the control group, and the DCM subgroup with low let-7i levels was associated with poor clinical outcomes [12]. This consequently implies that let-7i might be a novel biomarker for clinical outcomes for patients with DCM (Figure 2). Besides DCM, hints also exist that let-7 participate in the cardiomyopathy caused by some drugs. Fu *et al.* found that let-7g was suppressed significantly in the doxorubicin-induced cardiomyopathy [42].

### 2.4. Let-7 in Arrhythmia

Excitability is a fundamental feature of cardiac cells. Cardiac excitability is conferred by heart electrical activities orchestrating by a matrix of ion channels and transporters, the trans-membrane proteins that control the movement of ions across the cytoplasmic membrane of cardiomyocytes. The interplay of ion channels maintain the normal heart rhythm. Channelopathies, disease caused by dysfunction of the ion channels, can render electrical disturbances predisposing to cardiac arrhythmias [43]. The involvement of miRNAs in arrhythmia has been recognized recently [26,44–46]. Let-7 is the fifth most abundant miRNA in myocardium, and is predicted to regulate main cardiac conduction by targeting SCN5A (Nav1.5 for intracellular conduction), GJC1 (Cx45 for intercellular conduction), GJA1 (Cx43 for intercellular conduction) and CACNA1C (Cav1.2 for the characteristic long plateau of the cardiac action potential and excitation-contraction coupling), resulting in the *I*_Na_, *I*_Ca_ reduction and eventually account partly for the conduction damage and arrhythmias (Figure 2) [26]. Furthermore, it has been shown that by affecting Na^+^ and Ca^2+^ channels, let-7f participate in arrhythmias caused by MI [26].

### 2.5. Let-7 in Angiogenesis

**Figure 3:**
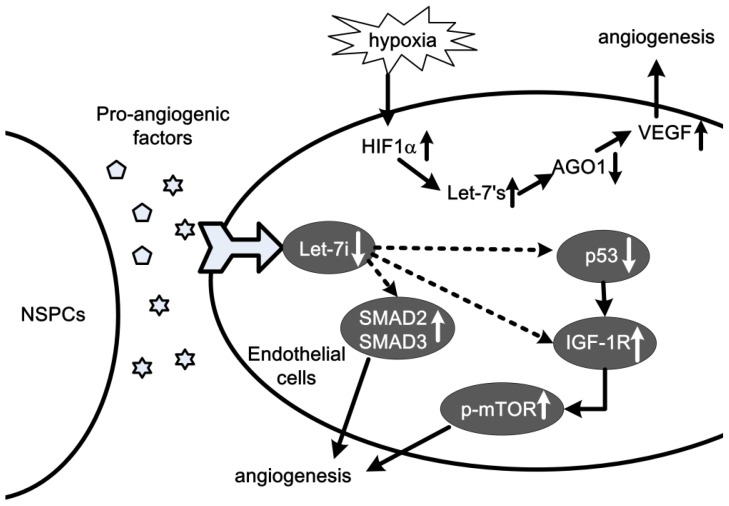
Roles and signaling pathways of let-7 in hypoxia- and neural stem/progenitor cells (NSPCs)-induced angiogenesis. Hypoxia treatment on endothelial cells (ECs) induced angiogenesis through hypoxia-inducible factor 1α (HIF1α)/let-7s/(AGO1)/VEGF signaling pathway. In ECs co-incubated with NSPCs, pro-angiogenic factors released by the NSPCs induced angiogenesis through SMAD2 (SMAD3), and p53/IGF-1R/p-mTOR signaling pathways.

In vascular development and homeostasis, the formation of new blood vessel through the process of angiogenesis is critical. Endothelial cells are key regulators of different aspects of vascular biology, including the formation of new blood vessels (angiogenesis). Aberrant angiogenesis leads to numerous disorders, such as cancer and ischemia. Several studies have reported the important roles of miRNAs in regulating ECs in angiogenesis, e.g., miR-126, miR-17~92 cluster, miR-378 and miR-296 [47–51]. Let-7 has been found highly expressed in HUVECs [52], and exhibited to play critical roles in angiogenesis. The effects of let-7 on angiogenesis were first identified by Dicer and Drosha knockdown method. After Drosha and Dicer knockdown, let-7a, let-7b, let-7c, let-7f and let-7g were reduced by more than 30%. The reduction of let-7f has been verified to significantly impair the sprout formation [52]. Similar effects were found for let-7a [53] and let-7b [54]. Although the detailed signal network is yet not determined, many angiogenesis-related factors have been shown to participate in let-7 regulated angiogenesis, such as thrombospondin-1 [53,54], thrombospondin-2 [55], TIMP-1 [54], Nrp-2 and c-Met [56], TEK/Tie-2, KDR/VEGFR2 and Tie-1 [53]. A very recent study found the hypoxia-inducible factor 1α (HIF1α)/let-7/argonaute 1(AGO1)/VEGF signal pathway in hypoxia-induced angiogenesis. HIF1α is a key transcription factor, which up-regulates the expressions of let-7, and let-7 targeted AGO1, resulting in the translational desuppression of *VEGF* mRNA as well as angiogenesis [11] (Figure 3). These findings provide evidence for an angiogenic pathway involving let-7 that targets *AGO1* and suggests that this pathway may be a suitable target for anti- or pro-angiogenesis strategies. Interestingly, compared with other studies [53,54], an inverse regulative direction of let-7’s was found in this research. This conflict might indicate a complex mechanism of let-7 on angiogenesis, which still need further studies.

Other interesting findings about let-7 suggest that it might participate in cell to cell actions in angiogenesis. For instance, in glioma cells co-incubated human brain microvascular endothelial cells, and in neural stem/progenitor cells (NSPC) co-incubated ECs also, let-7 members display decreased levels [49,57]. Glioma cells and NSPC are considered to promote tumor or brain angiogenesis [58]. Although the contribution of let-7 on glioma cell-induced tumor angiogenesis is still to be clarified, the effects of let-7i on NSPC-induced brain angiogenesis may partly be obtained through the SMAD2/SMAD3 and p53/IGF-1R/p-mTOR signaling pathways [57] (Figure 3).

### 2.6. Let-7 in Atherogenesis and Coronary Artery Disease

**Figure 4:**
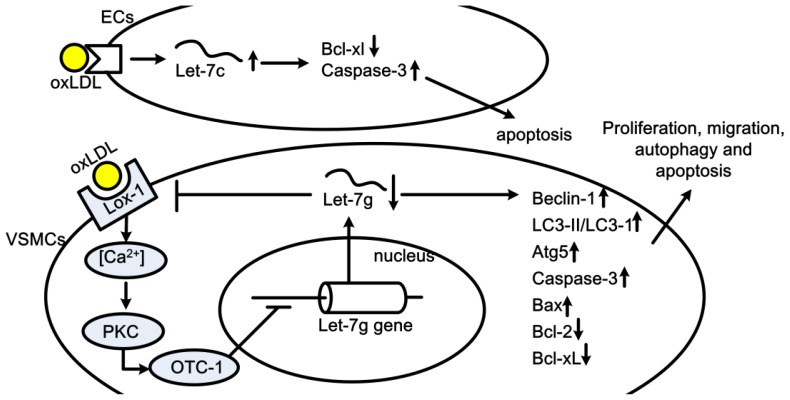
Roles of let-7 in oxLDL induced VSMCs and ECs apoptosis, proliferation, migration and autophagy. OxLDL induces EC apoptosis via let-7c and subsequent Bcl-xl and Caspase-3. In oxLDL treated VSMCs, oxLDL suppresses let-7g expression via LOX-1/Ca^2+^/PKC/OTC-1 signaling pathway, the let-7g then targets on Beclin-1, LC3-II (LC3-I), Atg5, Caspase-3, Bax, Bcl-2, and Bcl-xl, and regulates the proliferation, migration, autophagy and apoptosis of VSMC.

Atherosclerosis (AS) is a chronic multifactorial inflammatory disease with a high prevalence and has become the major cause of death worldwide. The principal clinical manifestations of atherosclerosis are represented by coronary artery disease (CAD), acute myocardial infarction (AMI), cerebral stroke and peripheral vascular disease. The vascular EC injuries are considered to be the initial step of AS, and subsequent leukocytes invasion, vascular smooth muscle cell proliferation and foam cell formation all contribute to the atherogenesis [59–61]. Let-7s are abundantly expressed miRNAs in both ECs and VSMCs. Their roles on AS and CAD have also been demonstrated recently by several studies *in vivo* and *in vitro* [10,62,63]. Let-7g has shown to be reduced in oxLDL treated VSMCs, and this reduction may partially be responsible to the oxLDL-induced cell proliferation and migration [63], autophagy and apoptosis [10]. Lectin-like oxidized LDL receptor-1 (LOX-1), a surface scavenger receptor for oxLDL, has been identified to be the target of Let-7g. The downstreams of LOX-1, such as Ca^2+^, PKC and OCT-1, are subsequently impacted by let-7g. On the other hand, the transcription factor, OCT-1, also modulates the transcription of let-7g gene. Thus, a negative feedback loop exists between LOX-1 and let-7g [63]. Other factors related to VSMCs functions, such as ROS, autophagy-related proteins (expression of beclin-1, LC3-II/LC3-I ratio and Atg5) and apoptosis-related proteins (expression of caspase-3, Bax, Bcl-2 and Bcl-xL), are involved in the effects of let-7g [10]. Nonetheless, in oxLDL-induced EC apoptosis, let-7c seems to be up-regulated, and this up-regulation promotes EC apoptosis by targeting anti-apoptotic protein Bcl-xl [62]. Thus, let-7g might be an anti-atherogenesis factor and let-7c be a pro-atherosclerosis factor (Figure 4).

In *in vivo* studies, let-7g levels were found reduced both in high-fat diet mice and hypercholesterolemia humans, in accordance with *in vitro* results [60]. Let-7d and let-7i levels are down-regulated in CAD patients [27,64], and let-7s are up-regulated in patients suffering atherosclerotic abdominal aortic aneurysm (AAA) [65]. Intriguingly, there is a negative correlation between let-7i and TLR4 levels in CAD patients, similar to results from *in vitro* reports [24]. Because TLR4 signal is involved in pro-inflammatory cytokines release and contribute to CAD and heart failure [66], let-7i may be a participator in CAD via TLR4. Via influencing the let-7i expressions, atorvastatin treatment markedly down-regulates TLR4 signal in CAD patients, possibly contributing to the beneficial effects of atorvastatin on these patients [27].

Neointimal formation is a common pathological lesion in various cardiovascular diseases, such as atherosclerosis, coronary heart diseases and transplantation arteriopathy. In the angioplasty neointimal, let-7s are down-regulated dramatically [15]. Though the details of let-7’s effects on angioplasty are still unclear, miRNAs may be new therapeutic target for proliferative vascular diseases such as atherosclerosis, postangioplasty restenosis, transplantation arteriopathy, and stroke.

### 2.7. Let-7 in Hypertension

Essential hypertension is a cause for many diseases, such as stroke, MI, and CHF. Several studies have shown the relationship between miRNAs and hypertension [67–69]. However, the relationship between let-7 and hypertenstion had bot been reported until very recently. Li *et al.* [70] compared the miRNA expressions in the plasma samples from hypertensive patients and healthy control subjects. The expressions of 27 miRNAs were found changed, among with 9 miRNAs were up-regulated, and 18, down-regulated. Particularly, let-7e was found to be regulated up to 1.7 folds of control (*p* < 0.0001). Interestingly, the cellular origin of plasma let-7e seems to be partially from endothelial cells rather than endothelial progenitor cells (EPCs). It was found that let-7e levels in ECs from hypertensive patients were higher than those from control subjects, and in patient plasma samples, this similar expression trend was also observed. On the other hand, the changes in let-7e levels in EPCs were opposite to those in plasma. No differences in let-7e levels in peripheral blood mononuclear cells were observed between the hypertensive patients and control subjects. The targets and the role of let-7e in hypertension are still open for further exploration.

## 3. Let-7 in Heart Development

The roles of let-7 on heart development were evaluated by fetal mouse heart, namely, at 4 key time-points [embryonic (E) day E12.5, E14.5, E16.5 and E18.5] during its development. In the longer development group, several members of the let-7 (let-7a/7d/7e/7f) were up-regulated. Bioinformatic analysis predicts five cardiac development related genes (*FOXP1*, *TBX5*, *HAND1*, *AKT2* and *PPARGC1A*) to be the targets of let-7a/7d/7e/7f (Figure 2). These findings indicate that let-7 might participate in normal heart development, and the aberrant expression of let-7 might result in congenital heart disease [30].

## 4. Let-7 in Cardiovascular Differentiation of Embryonic Stem Cells

It is well known that postnatal hearts are unable to repair following injuries. With more and more people becoming victims of heart failure worldwide, the embryonic stem cells (ESCs) differentiated cardiovascular has gained great attentions recently. During the process of human ESC differentiation into myocardial precursors and cardiomyocytes (CMs), several miRNAs are specifically regulated, and may play important role in supporting the differentiation or provide biomarkers for the stem cell-derived cardiomyocytes. Wong *et al.* [71] examined >900 miRNAs from human ESC to myocardial precursors on day 8 and 14 of the differentiation, and identified 95 miRNAs on day 8 and 67 miRNAs on day 14. The results exhibit changes in expressions whose magnitudes are more than two folds, and miR-1, miR-133, let-7 as well as miR-125b are among these miRNAs.

Interestingly, in ESCs at different stages of differentiation ESCs, such as undifferentiated hESC, hESC-derived (hE-), fetal (hF-) and adult (hA-) ventricular (V) CMs, let-7s are differently expressed. They are expressed higher in hE-, hA-, and hF-VCMs than in undifferentiated ESCs [28]. Introduction of let-7s can suppress self-renewal in *Dgcr8*^−/−^ ESCs and induce differentiation [29]. These results indicate that let-7 might make important contribution in cardiavascular-oriented ESCs differentiation processes.

In the cardiovascular differentiation of ESCs, a complex network exists among various miRNAs. For example, miR-125b has been shown to regulate let-7 expression via targeting lin-28, an inhibitor of let-7 [71].

Induced pluripotent stem (iPS) cells are considered to be similar to ESCs [72]. Compared to those in iPS, let-7s are abrogated in iPS-derived cardiomyocytes, and iPS is demonstrated to be effective to cure acute myocardial infarction (AMI) in mice [73].

## 5. Circulating Let-7 as a Mediator of Intercellular Communication and as a Biomarker of Cardiovascular Disease

MiRNAs has been recently revealed to be secreted by donor cells and can influence the gene expressions of recipient cells. Although the secretory mechanisms and its impacts are yet to be explored, the secreted miRNAs have recently been considered as an emerging form of intrercellular communication. Since both actively secreted miRNAs and passively leaked miRNAs can be released in a stable, cell-free form to circulation, they are also called circulating miRNAs [74]. miRNAs can enter the circulation through three pathways: (i) passive leakage from broken cells; (ii) active secretion via microvesicles, including exosomes and shedding vesicles; and (iii) active secretion in conjunction with the RNA-binding protein high-density lipoprotein (HDL) [73]. The circulating let-7b has been reported to be secreted by mouse preadipocyte cell line (3T3-L1) in the form of microvesicles, and might be transferred into macrophages. However, the biological functions of intercellular transfer of miRNAs from donor cells to recipient cells remain unknown [75,76].

Recently, the circulating miRNAs have been reported as potential biomarkers for various pathologic conditions [77–79]. In AMI patients, the authors found let-7b being dramatically inhibited in 4 to 12 h of the onset of AMI, and reached its expression peak at 8 h, which was similar to the peak time of cardiac troponin I (cTnI), a maker with high specificity for cardiac injury. Results of the receiver operating characteristic (ROC) curve analyses suggest let-7b be of significant diagnostic value for AMI [80]. Thus, in addition to cTnI, let-7b might be a potential combined diagnosis marker to further confirmation of the AMI, since the down-regulation of let-7b is consistent with the up-regulation of cTnI, and the change of let-7b can be detected in a relative short time after onset of AMI. However, further studies are necessary to determine whether or not let-7b can be used as a prognostic indicator. In DCM patients, the expression of let-7i is significantly down-regulated compared with the healthy control group. Further studies discovered that the DCM subgroup with low let-7i levels yielded poor clinical outcomes [12]. This may imply that let-7i be a novel biomarker for clinical outcomes in DCM patients. However, in atherosclerosis patients, let-7f is observed significantly increased in tissue samples, but not in blood samples. Because of the difficulty to obtain tissue samples, let-7f shows little value as a biomarker. However, it may be used as a therapeutic target [81].

## 6. Conclusion and Perspective

**Figure 5:**
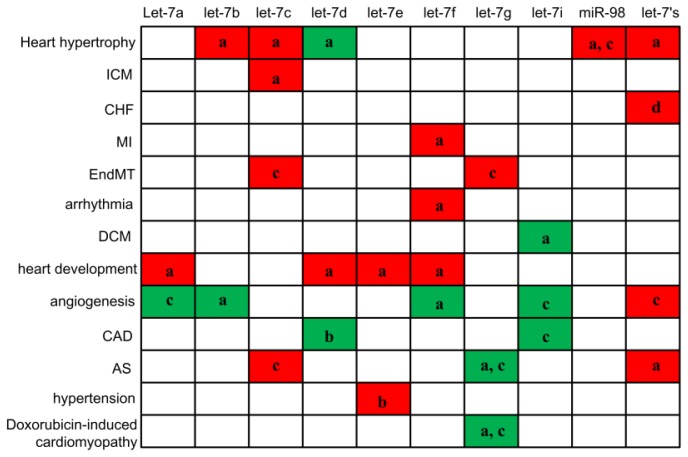
Modulation of let-7 in different cardiovascular diseases. **Red** color indicates let-7s that are significantly up-regulated, **green** color indicates let-7s that are significantly down-regulated. ICM: ischemic cardomyopathy; CHF: congestive heart failure; MI: myocardial infarction; DCM: dilated cardiomyopathy; CAD: coronary artery disease; AS: atherosclerosis; a: tissue samples; b: circulation (plasma) levels; c: cells levels, d: bioinformatics studies.

Both *in vivo* and *in vitro* studies have revealed the aberrant expression of let-7 in diverse cardiovascular diseases, heart development and cardiovascular differentiation, implying they may play important roles in these processes. It should be noted that the expression trends of specific let-7 members are disease- and organ-specific. For example, in most heart diseases (heart hypertrophy, ICM, CHF, MI, arrhythmia) and in heart development, let-7s are up-regulated, while in most vascular diseases (angiogenesis, CAD) they are down-regulated. For the expression of a particular group of let-7s, there is a particular pathological process associated. For example, in heart hypertrophy, let-7b/7c/miR-98 are up-regulated and let-7d is down-regulated. These signature patterns can provide aids in the diagnosis and prognosis of human diseases, as evidenced by recent studies on cardiovascular diseases (Figure 5, Table 1) [11,80]. However, better understandings on the functions of let-7s are needed to determine whether let-7s can be potential therapeutic targets or not. Only a handful of targets for let-7s have by now been discovered, such as TLR4, lox-1, Bcl-xl and AGO1. It is critical to identify their gene targets and signaling pathways responsible for their cardiovascular effects in future studies. Moreover, how the expressions of let-7s are regulated in cardiovascular diseases is currently unclear. Studies on the regulation of these aberrantly expressed let-7s are necessary and promising.

Cardiovascular diseases are the consequences of combined action of many tissue and cells including cardiomyocytes, endothelial cells, white blood cells, and smooth muscle cells. Since the secreted miRNAs have been revealed to play important roles in intra- and inter-cellular communications and multiple pathological processes [70], the identification of the biological roles of circulating let-7s should be done. In summary, the ultimate goal of the research orientation is to obtain let-7s based therapeutic and diagnostic strategies for cardiovascular diseases, to realize roles of let-7s in cardiovascular development, functions, and dysfunctions and to provide new insights into these disorders.

## Figures and Tables

**Table 1:** Synopsis of let-7 in cardiacvascular diseases, heart development and cardiavascular differentiation from stem cells.

Let-7 member	Up-/downregulation during cardiovascular diseases	Tissue or cell samples used	Predicted (in *italic*) or confirmed targeted genes	References
Let-7a	Heart development ↑	Mouse fetal hearts	*(FOXP1, TBX5, HAND1, AKT2, PPARGC1A)*	[30]
	Angiogenesis ↓	Human endothelial cells	*Nrp-2*	[53]
Let-7b	Heart hypertrophy ↑	Mouse heart	Undetected	[21]
	Angiogenesis ↓	Mouse ovary vessel	TIMP-1 *c-Met*	[54]
Let-7c	Heart hypertrophy ↑	Mouse heart	Undetected	[21]
	ICM ↑	Human left ventricular	Undetected	[22]
	EndMT ↑	Mouse cardiac endothelial cells	Undetected	[40]
	AS ↑	human endothelial cells	Bcl-xl	[62]
Let-7e	Heart development ↑	Mouse fetal hearts	*(FOXP1, TBX5, HAND1, AKT2, PPARGC1A)*	[30]
	Hypertension ↑	Human plasma samples (the origin is endothelial cells)	Undetected	[70]
Let-7f	Arrhythmia ↑	Rat hearts	Undetected	[26]
	MI ↑	Rat hearts	Undetected	[26]
	Heart development ↑	Mouse fetal hearts	*(FOXP1, TBX5, HAND1, AKT2, PPARGC1A)*	[30]
	Angiogenesis ↓	Human endothelial cells	*thrombospondin-1, TSP-2*	[52]
Let-7g	EndMT ↑	Mouse cardiac endothelial cells	Undetected	[40]
	AS ↓	VSMCs, and mice aorta	Lectin-like LDL receptor 1	[63]
	Doxorubicin-induced cardiomyopathy ↓	Rat hearts and cardiac myocytes	Undetected	[42]
Let-7i	DCM ↓	Endomyocardial biopsy tissues	Toll like receptor 4	[24]
	Angiogenesis ↓	Human endothelial cells	*IGF-1R*	[57]
	CAD ↓	THP-1 cells and patient blood monocytes	Toll like receptor 4	[64]
MiR-98	Heart hypertrophy ↑	Mouse heart, and cardiac myocyte	Thioredoxin 1	[34,35]
Let-7’s	Heart hypertrophy ↑	Mouse heart	Undetected	[34]
	CHF ↑	Bioinformatics studies	Undetected	[25]
	Atherosclerotic AAA ↑	Human aortic aneurysm	Undetected	[65]
	Angiogenesis ↑	Human endothelial cells	Argonaute 1	[11]
	ESC differentiation to myocardiac cells ↑	ESCs and ESC-derived cardiomyocytes	Undetected	[28,29,71]
	iPS differentiation to cardiomyocytes ↓	iPS cells and iPS-derived cardiomyocytes	Undetected	[73]
